# Understanding Behavioral Influences on Eating Disorders and App Engagement to Inform Eating Disorder App Development: Qualitative Online Focus Groups With Adults With Lived Experience

**DOI:** 10.2196/79328

**Published:** 2026-01-28

**Authors:** Pamela Carien Thomas, Sarah Rowe, Kristina Curtis, Rachel Perowne, Pippa Bark

**Affiliations:** 1 Epidemiology and Applied Clinical Research Department University College London London, England United Kingdom; 2 Applied Behaviour Change Leamington Spa United Kingdom; 3 Department of Clinical, Educational and Health Psychology University College London London United Kingdom; 4 UCL Cancer Institute University College London London United Kingdom

**Keywords:** eating disorders, mobile apps, digital health, behavior change, COM-B model, capability, opportunity, motivation—behavior, lived experience, qualitative research

## Abstract

**Background:**

Eating disorders (EDs) are severe mental health conditions driven by psychological, social, and emotional factors and have the highest mortality rate of any psychiatric disorder. Although evidence-based, theory-driven behavior change interventions are the gold standard, access to treatment remains limited. Digital interventions, such as apps, may offer accessible support for individuals with mild to moderate EDs; however, their development has rarely been guided by systematic behavior change frameworks. Consequently, many interventions inadequately target the mechanisms underlying ED behaviors and commonly lack involvement of people with lived experience.

**Objective:**

This study aimed to identify priority behavioral change targets for ED apps by capturing lived experience perspectives on the psychological, behavioral, and contextual factors maintaining disordered eating and driving app engagement. Using the capability, opportunity, motivation—behavior (COM-B) and theoretical domains framework (TDF), we mapped these determinants to identify where and how apps can most effectively enhance capability, opportunity, and motivation.

**Methods:**

In total, 6 small focus groups (2-5 participants per group) were conducted with 13 female and 5 male adults, including minority ethnic backgrounds, living in the United Kingdom with lived experience of an ED. Discussions explored (1) the psychological, social, and environmental determinants underpinning participants’ disordered eating behaviors and (2) the behavioral and contextual mechanisms influencing engagement with an ED app. A hybrid deductive-inductive thematic analysis was performed using the COM-B model and the TDF. Themes were mapped onto evidence-based behavior change techniques using the Theory and Techniques Tool.

**Results:**

This study identified clear behavior change targets for digital ED interventions, identifying requirements in 5 of 6 (83%) COM-B domains and 13 of 14 (93%) associated TDF domains for changing maladaptive ED behaviors and 5 of 6 (83%) COM-B and 12 of 14 (86%) TDF domains for sustaining app engagement. Although social support and emotional regulation were key influences, less commonly targeted domains, such as social or professional role and identity and belief in capabilities, emerged as powerful drivers in this population. Crucially, it demonstrated that effectiveness depended not only on which behavior change techniques were included but also on how they were implemented, as poorly delivered techniques can undermine engagement and exacerbate symptoms. Sex and cultural background moderated almost every domain, highlighting the necessity of personalized, adaptive delivery and the inadequacy of one-size-fits-all approaches.

**Conclusions:**

As the first study to apply the COM-B and TDF frameworks to both disordered eating behaviors and app engagement, it identifies previously overlooked behavioral mechanisms and design pitfalls, including how poorly delivered techniques can undermine recovery. It provides a practical blueprint for developing safer, more personalized, and behaviorally effective ED apps. Significant work is needed to advance apps in line with these recommendations, supported by ongoing collaboration with diverse people with lived experience.

## Introduction

### Background

Eating disorders (EDs) are severe mental health conditions with the highest mortality rate of any psychiatric disorder [1] and are associated with significant psychological, physical, and emotional consequences [2]. Globally, EDs are estimated to affect up to 70 million people, with prevalence rates rising over recent years [3]. Despite the substantial burden of disease on individuals [4] and the health care system [5], access to treatment remains a significant challenge [6]. A US study revealed that 85.9% of individuals who had screened positive for an ED never received treatment [7]. As EDs rarely resolve spontaneously and often worsen over time, early intervention is essential [8]. EDs affect people of both sexes and all ages, ethnicities, and backgrounds, with similar prevalence rates of disordered eating in male and female individuals and in minority ethnic groups compared to White individuals [9,10]; yet, these groups experience more limited access to treatment [11,12].

Understanding the behavioral mechanisms that underpin EDs is critical for developing digital interventions, such as apps, that can effectively modify these behaviors. Existing research has highlighted key mechanisms contributing to ED behaviors, including rigid cognitive control, affect regulation difficulties, perfectionism, and negative body image [13-15]. However, it remains unclear how these mechanisms translate into the specific capabilities, opportunities, and motivations that drive restricting, bingeing, or purging in everyday life. Studies exploring these behavioral determinants through in-depth discussions with people with lived experience remain limited, and most have focused on predominantly White, female samples [16], overlooking potentially distinct influences affecting male individuals and individuals from minority ethnic groups [17,18]. To date, no study has systematically mapped these behaviors using behavior change frameworks such as capability, opportunity, motivation—behavior (COM-B) [19] and the theoretical domains framework (TDF) [20], leaving digital intervention development without a clear, theory-informed basis for targeting the behaviors that need to change [21,22].

Despite the proliferation of digital ED interventions, systematic reviews have consistently highlighted a significant gap in the incorporation of evidence-based behavior change strategies. Most commercially available apps offer minimal content, such as basic mood or food tracking and generic motivational messages, with little structured cognitive or behavioral support [23-27]. Even among research-based apps [28,29], key domains, including structured social support, environmental restructuring, and culturally tailored resources, remain underdeveloped. Usability issues [30] such as those surrounding food logging [16,31] suggest that the way behavior change components are implemented can undermine engagement. Importantly, while some usability challenges mirror those seen in broader mental health apps (eg, lack of personalization), others are unique to ED interventions, such as food tracking or numeric logging that can exacerbate perfectionism and shame [32]. These gaps highlight the need for systematic, theory-driven app development that maps behavioral mechanisms to intervention features and involvement of people with lived experience in the design process [33].

Digital interventions, particularly apps, offer accessible, flexible, and anonymous support, making them particularly suitable for individuals who might not otherwise seek help [34]. Systematic reviews suggest that digital ED intervention can be effective [27,35-37]; however, engagement (defined as uptake, sustained use, and attrition) remains a significant barrier to realizing this potential [26,36,38]. Existing self-guided apps often lack technological sophistication, meaningful interactivity, personalization, or social components, and no specific design features have been consistently associated with lower dropout [39]. Techniques effective in face-to-face therapy may require careful adaptation to maintain engagement in self-guided digital interventions. Qualitative research has shown that poor usability, perceived irrelevance, and lack of personalization are key drivers of early disengagement [16,40]. Although previous research has provided valuable insights into general usability issues and surface-level preferences, no prior study has comprehensively applied a theory-driven (COM-B or TDF) framework to map the behavioral determinants that drive sustained engagement with ED-specific apps from the perspective of people with lived experience. This gap is especially pronounced for underrepresented groups, including male individuals [41] and individuals from minority ethnic backgrounds [11,12], whose needs are frequently overlooked in the design of both traditional [42] and digital ED interventions [16]. Addressing these gaps is essential not only for inclusivity but also to ensure that digital ED interventions are acceptable, effective, and equitable across diverse populations.

### Theoretical Framework

**Figure 1:**
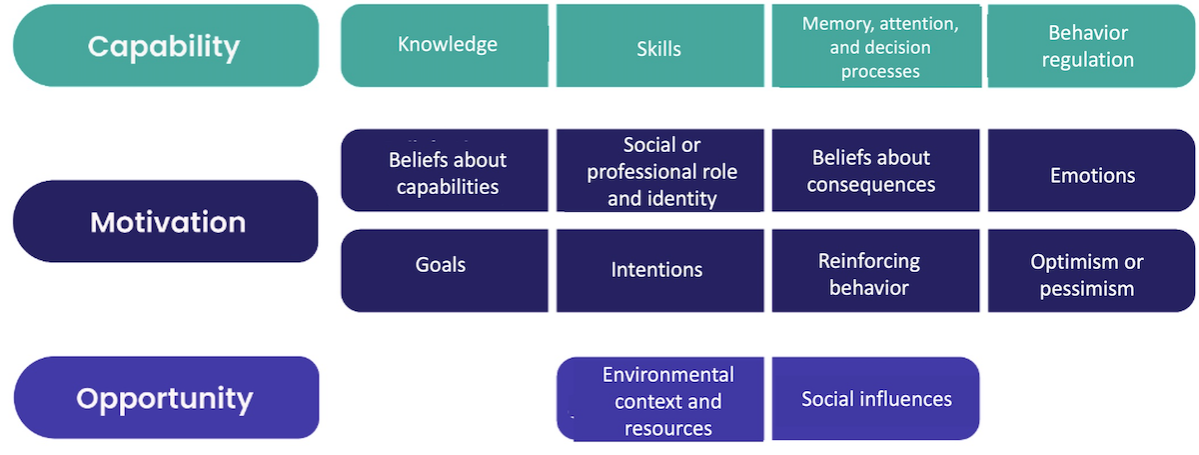
The capability, motivation, and opportunity model and the theoretical domains framework.

Behavioral models such as the COM-B model [19] and the TDF [20] provide a systematic approach to understanding the drivers of behavior and informing the design of effective interventions. The COM-B posits that behavior change depends on the interaction of 3 factors: capability, opportunity, and motivation. The TDF is a comprehensive framework that consolidates 14 domains (eg, knowledge, skills, beliefs about capabilities, and social influences) to identify barriers and facilitators of behavior change, drawing from various psychological and behavioral theories [22]. It is closely related to the COM-B model, providing a detailed breakdown of the components within capability, opportunity, and motivation (Figure 1) [37], allowing for a more granular analysis of the factors influencing behavior. This connection enables the TDF to operationalize COM-B by offering specific constructs that can be targeted when designing interventions to address identified behavioral determinants. This methodology is a crucial step toward designing useful interventions [43].

These frameworks have been applied in various health contexts, including physical activity and smoking cessation [22,44,45], to identify the underlying mechanisms of behaviors and design interventions targeting these mechanisms. No systematic analysis has applied the COM-B model and TDF to identify specific behavioral domains in EDs, which has limited the development of targeted ED interventions [19,20].

### Rationale and Study Aims

Incorporating behavior change theory into app development has been shown to improve user effectiveness and engagement [46]. While systematic reviews highlight the value of specific behavior change techniques (BCTs) [27], there is limited evidence regarding how these techniques align with the behavioral influences driving restrictive, bingeing, or purging behaviors or how they can be operationalized in digital contexts.

This study addressed this gap by exploring the behavioral influences underlying maladaptive ED behaviors and app engagement among people with lived experience, using the COM-B and TDF frameworks, and systematically mapping these influences to BCTs via the BCT Taxonomy v1 [47]. The study aimed to inform the design of theory-driven, user-informed digital interventions that are effective, inclusive, engaging, and responsive to diverse needs.

The objectives of this study were to identify the key behavioral, psychological, and contextual factors maintaining maladaptive ED behaviors; capture perspectives from male and female adults across different ethnic backgrounds to ensure inclusivity; identify the behavioral and contextual determinants of engagement with ED apps; and map identified behavioral influences to BCTs and consider how they can be prioritized and implemented sensitively in digital contexts.

## Methods

### Study Design

Small focus groups (FGs) of people with lived experience of EDs ensured that all had the opportunity to share their perspectives while fostering a “safer” and more intimate setting that encouraged active and meaningful contributions [48]. The number of sessions was informed by the point of data saturation of the key themes [48]. The use of online FGs allowed for the inclusion of diverse voices from geographically dispersed participants, which was relevant, given the goal to make treatment more widely accessible.

The COREQ (Consolidated Criteria for Reporting Qualitative Research) checklist [49] ensured that the procedures, analysis, and interpretation were rigorous (Multimedia Appendix 1).

### Recruitment

The primary recruitment channel was First Steps ED, a specialist ED support service, which accessed individuals with relevant lived experience. This was supplemented by targeted advertisements posted on social media platforms, specifically Facebook and X (formerly known as Twitter). Since most apps are targeted mainly at the Western female population, we explicitly aimed to recruit a diverse sample, including a range of ages, male participants, and individuals from minority ethnic backgrounds.

In response to inauthentic or bot-generated responses, a structured screening protocol was developed in consultation with a second qualitative researcher (PB) and university IT support to ensure genuine, eligible participants. Of 95 emailed expressions of interest, 75 were excluded due to suspected inauthentic responses (eg, verbatim or near-verbatim responses and timestamp clustering; Multimedia Appendix 2). Flagged cases were independently reviewed by 2 researchers (PCT and PB), with exclusion requiring consensus.

The remaining 20 respondents were sent a screening form to assess eligibility by detailing their ED history, including self-reported diagnosis (if any), treatment history, and current or recent ED behaviors (eg, binge eating, purging, or restriction ≥1 time per week for ≥3 months, per *DSM-5* (*Diagnostic and Statistical Manual of Mental Disorders* [Fifth Edition]) subthreshold criteria). A formal diagnosis was not required, aligning with the study’s focus on lived experience. Interested individuals were emailed, and those meeting the inclusion and exclusion criteria (Textbox 1) were sent study information and consent materials. A sample of 20 was considered adequate to provide thematic depth and diversity in perspectives, appropriate for the study’s qualitative co-design approach [50]. Recruitment materials (advertisements, consent forms, and screening survey) were provided in English only, reflecting the UK-based sample and the FG language.

**Inclusion and exclusion criteria for online focus groups with people with lived experience.**

**Inclusion criteria**

- Adults with eating disorders or eating disorder behaviors or symptoms (aged 18 years and older).
- Adults who were interested in getting support for their eating disorder or disordered eating problems.
- Adults who had experienced an eating disorder or disordered eating and were in recovery.

**Exclusion criteria**

- Receiving treatment for eating disorders at the time of the study.
- Had been hospitalized for an eating disorder within the last 3 years.
- Underweight at the time of the study (classified as a BMI less than 18.0 kg/m^2^).
- Did not feel or present in a fit mental state to participate.

### FG Procedure

A semistructured topic guide was designed to cover key behavioral domains relevant to intervention development. The structure and content were developed and piloted with Patient and Public Involvement from First Steps ED. It was structured into 3 sections: ED experiences and digital tools, influences on ED behaviors (subdivided into psychological and emotional factors, social and environmental influences, and motivational factors), and engagement with support apps (Multimedia Appendix 3).

FGs took place in January and February 2024 and were held online in the evening. While participants had no prior relationship with the researcher, rapport was established through presession email exchanges that included session outlines and answered queries. Participants were aware of the researcher’s role and the study’s aim to inform the development of a supportive ED app.

### Reflexivity Statement

The lead researcher (PCT), a female PhD candidate trained in qualitative methods, conducted and analyzed the online FGs. She remained reflexive about potential biases, including a belief in the value of digital tools for addressing service gaps, which could influence questioning, coding, or interpretation. To mitigate this, she actively explored diverse views, including critical perspectives on ED apps. Coding and analysis were regularly discussed with the second supervisor (PB), who provided independent input and supported the development of balanced, grounded themes through ongoing analytical debriefing.

### Ethical Considerations

This study received ethics approval from University College London Research Ethics Committee (UCL Ethics: 23943/001). The research adhered to ethical guidelines for qualitative studies involving human participants. Eligible participants who met the inclusion criteria received a detailed participant information sheet outlining the study’s purpose, procedures, potential risks, and the voluntary nature of participation. Informed consent and baseline demographic data were collected via REDCap (Research Electronic Data Capture) [51], a secure, web-based platform hosted by University College London. Participant data were stored on encrypted, password-protected systems accessible only to the research team. All transcripts were fully anonymized, with identifying information removed prior to analysis. During FGs, participants used first names only to reduce identifiability. Quotations presented in this manuscript were reviewed to ensure anonymity with no personally identifiable details. To address the sensitive nature of EDs, safeguarding measures were implemented. Interview questions were piloted with a Patient and Public Involvement committee to ensure sensitivity and relevance. The primary researcher (PCT) was a trained facilitator with experience in motivational interviewing and previous volunteering with an ED charity. Immediate safeguarding support was available during and after sessions by an on-call safeguarding lead at First Steps ED. In line with Health Research Authority guidance [52], participants received a US $34 voucher as compensation for their time and input, ensuring fairness without undue inducement.

### Data Analysis

All FG discussions were video recorded and automatically transcribed using Microsoft Teams, with transcripts subsequently checked for accuracy and anonymized. Transcripts were imported into NVivo (version 14; Lumivero) for analysis. A hybrid deductive-inductive thematic analysis was conducted using the COM-B model and the TDF as the primary organizing structure. All transcripts were first deductively coded line-by-line in NVivo (version 14) by the first reviewer (PCT) using COM-B components and the 14 TDF domains. To calibrate coding and enhance reliability, 2 researchers (PCT and RP), both trained in behavioral science, independently coded the first transcript together, refining code definitions through discussion. The second researcher (RP) then independently deductively coded 100% of the transcripts using the same framework. Discrepancies and emerging interpretations were documented in a Microsoft Excel spreadsheet and resolved in an iterative consensus meeting. A third reviewer (KC) was available to adjudicate unresolved disagreements. Final agreed inductive codes were grouped into subthemes.

Following the COM-B and TDF-informed analysis, the identified domains (eg, social or professional role and identity and emotions) were systematically mapped to corresponding BCTs using the BCT Taxonomy v1 [47]. This mapping process was primarily guided by the Theory and Techniques Tool [53], which provided evidence-based links between theoretical domains and BCTs in published intervention studies and indicated the strength of the evidence of each link. Where the Theory and Techniques Tool indicated strong or promising evidence for a link, the corresponding BCT was prioritized. In cases of conflicting evidence, weak evidence, or where multiple BCTs were plausible, final selection was determined by expert consensus within the multidisciplinary project team. Decisions were further refined to ensure contextual fit with study findings regarding day-to-day influences on ED behaviors and app engagement and the technical and design constraints of an app.

## Results

### Overview

Of the 95 initial recruitment responses, 75 inauthentic responses were excluded (Multimedia Appendix 2), and 2 withdrew prior to data collection due to conflicting commitments, resulting in 18 participants. In total, 6 online FGs (labeled FG A-F) were conducted, with an average of 3 participants (range 2-5) per group. FGs had a mean duration of 58.1 (SD 3.8; range 53.9-64.0) minutes.

### Participant Characteristics

In total, 13 of 18 (72%) adult female participants and 5 of 18 (18%) adult male participants took part, a ratio closely representative of the ED population [8]. A majority of 13 of 18 (72%) participants were White, and 5 of 18 (28%) were non-White including 2 of 18 (11%) Asian and 3 of 18 (11%) Black. In terms of age, 5 of 18 (28%) participants were aged 36 years or older, with the remaining 13 of 18 (72%) younger than 35 years.

Of the 18 participants, 14 (78%) reported engaging in dietary restriction, 10 (56%) reported overexercise as a symptom, 7 (39%) reported binge eating, and 6 (33%) reported purging. In total, 12 of 18 (67%) participants had received outpatient treatment, and 6 of 18 (33%) had received inpatient care. At the time of the study, 1 of 18 (6%) male participants was on a waiting list for treatment but was in a stable condition. A total of 5 of 18 (28%) participants reported never having received any form of treatment (Multimedia Appendix 4).

### Objective 1: Understanding Disordered Eating Behaviors

#### Overview

When discussing their ED behaviors, participants described behavioral influences across 5 of 6 COM-B domains (all domains excluding physical capability). There was consistency in the types of behavioral influences described; however, the relative importance of specific influences varied between individuals. These findings mapped to all areas of the COM-B, except for physical capability, which was not discussed, and to 13 of 14 (93%) TDF domains.

#### Psychological Capability

##### Overview

Results identified key psychological capability factors influencing disordered eating behavior, captured as themes and subthemes across COM-B and TDF domains with corresponding behavior change techniques (Table 1).

**Table 1:** Psychological capability factors influencing disordered eating behavior: capability, opportunity, motivation—behavior (COM-B) and theoretical domains framework (TDF) analysis.

COM-B domain	TDF domain	Subthemes	Behavior change technique
Psychological capability	Knowledge	Understanding eating disorder condition Importance of nutrition Connection between food and weight Relapses in recovery	4.1 Instructions on how to perform a behavior 4.2 Information about antecedents 5.1 Information about health consequences 5.3 Information about social and environmental consequences
Psychological capability	Skills	Cognitive skills (challenging negative thoughts) Need for coping skills (to manage urges) Interpersonal skills Meal planning	8.1 Behavioral practice or rehearsal 8.2 Behavior substitution 12.4 Distraction
Psychological capability	Behavioral regulation	Regulating eating Self-monitoring	2.3 Self-monitoring of behavior 2.4 Self-monitoring of outcomes of behavior 4.2 Information about antecedents 8.2 Behavior substitution
Psychological capability	Memory, attention, decision-making	Planning regular eating	1.4 Action planning 12.4 Distraction 13.2 Framing or reframing 11.3 Conserve mental resources

##### Knowledge

Participants often described a limited understanding of the triggers and consequences of their disordered eating, contributed to delayed recognition of symptoms, and reduced help-seeking. This lack of insight was accompanied by confusion, uncertainty, and a sense of helplessness about their experiences. As one participant explained, understanding the mechanisms underlying their behavior and what EDs really were “might have helped me recognize the problem earlier” (FG A, Participant 1).

Understanding and being able to recognize how emotional triggers contributed to disordered eating behaviors and perpetuated cycles was seen as crucial. The knowledge that relapse was a normal part of recovery was viewed as key to maintaining motivation and self-compassion. One participant reflected that reassurance about these “cycles” of recovery and relapse could help individuals feel less discouraged when setbacks occurred (FG B, Participant 1).

There was a common gap in understanding of the relationship between EDs and nutrition, including the importance of nutrition for proper bodily function. For some, acquiring nutritional knowledge empowered some to broaden their food choices and challenge avoidance behaviors: “I didn’t know that my body needed all of that different things and how it helps you function in different ways” (FG A, Participant 3).

Learning through experience about the relationship between food intake and weight stability helped participants re-evaluate previously held fears around eating. Realizing that weight remained stable after eating previously avoided foods was often described as a turning point in recovery:

When I started to eat and realize everything was OK, this really surprized me. After years of not ... I ate a sandwich, and nothing happened.

FG F, Participant 1

##### Skills

All 6 FGs identified deficits across cognitive, emotional, and interpersonal skill domains. Cognitive deficits manifested as rigid, binary food rules, with participants describing thoughts such as “all negative, catastrophizing ... this is good, this is bad” (FG A, Participant 2). These judgments drove daily food avoidance and ritualistic behaviors, reinforcing disordered patterns.

Distraction and mindfulness were applied to break automatic patterns. A self-taught urge-delay strategy, beginning with seconds and progressing to minutes, emerged as a critical step in disrupting immediate enactment: “I used to try and delay [the urge to purge] for a few seconds at first ... then longer and longer” (FG B, Participant 1).

While effective at building tolerance, this approach was effortful, and success was inconsistent, with one participant describing it as “white knuckling it” (FG C, Participant 2). This strategy frequently bridged to emotional regulation, often conceptualized via a “wave-riding” metaphor: “Until you learn to ride the wave of your emotions, it’s really hard to get further” (FG E, Participant 2).

The recurrence of this metaphor across 3 groups highlighted a common experiential model. Participants emphasized the importance of practical, in-the-moment tools, framing these skills as both recovery and prevention mechanisms: “I think if I had learned specific techniques for dealing with binge urges earlier, it could have prevented a lot of spiralling” (FG C, Participant 3).

Interpersonal skills focused on disclosure and boundary-setting. Participants requested scripted tools to support conversations with family members, noting that without such guidance, they tended to internalize distress, creating relational barriers that worsened symptoms: “Discussing it with someone ... eventually made it easier to explain what I was going through” (FG C, Participant 3).

Practical skills, including meal planning and preparation, were highly valued for structuring eating, reducing decision fatigue, and promoting regular intake. Meal planning emerged as the most requested skill, reducing food preoccupation and supporting consistent eating patterns.

Participants frequently described skill failure as a trigger for rapid symptom escalation. A single lapse, due to unavailable or unpracticed skills, could prompt shame, erode confidence, and lead to re-engagement with disordered behaviors: “I gave up for weeks because it felt pointless” (FG B, Participant 1).

This pattern highlighted the importance of structured skill development and real-time prompts to support adaptive behaviors at moments of need.

##### Behavioral Regulation

Participants commonly described difficulties with self-monitoring and behavioral regulation, particularly when using digital tools that framed tracking in quantitative or performance-driven terms. Features such as calorie or BMI logging and rigid tracking often intensified preoccupation with numbers and control, amplifying rather than reducing disordered cognitions and behaviors:

Logging food just makes me obsess over calories and numbers. It’s not helpful; it makes things worse.

FG E, Participant 2

A constant focus on metrics and inflexible reporting of food intake heightened anxiety and self-criticism, reducing perceived autonomy and self-efficacy. Conversely, participants viewed more reflective forms of self-monitoring, such as journaling emotional states or identifying behavioral patterns, as safer, more supportive approaches. These features were described as increasing insights, emotional awareness, and self-compassion, enabling participants to regulate their behaviors in a more flexible and supportive manner.

##### Memory, Attention, and Decision-Making Processes

Practical organizational skills such as meal planning, shopping routines, and simple food preparation were key enablers for regular eating and reduced avoidance. Establishing a basic structure around eating was described as especially helpful for regaining consistency after periods of restriction:

Planning meals in a very simple way ... because I was skipping meals ... starting with that to make sure I had something for breakfast, then at a coffee or something as a snack, then a meal for lunch, then a snack, and then a dinner.

FG A, Participant 2

#### Opportunity (Environmental and Social)

##### Overview

Results identified key environmental and social opportunity factors influencing disordered eating behavior, captured as themes and subthemes across COM-B and TDF domains with corresponding behavior change techniques (Table 2).

**Table 2:** Environmental and social opportunity factors influencing disordered eating behavior: capability, opportunity, motivation—behavior (COM-B) and theoretical domains framework (TDF) analysis.

COM-B domain	TDF domain	Subthemes	Behavior change technique
Environmental opportunity	Environmental context and resources	Unstructured mealtimes Access to trigger foods Cultural norms around body image and food	12.1 Restructuring the physical environment 7.1 Prompts or cues 5.3 Information about social and environmental consequences 13.2 Framing or reframing
Social opportunity	Social influences	Befriending or peer support (advice, role models) Community or opportunity to share experiences Trusted people to talk to Negative impact of social pressures (family, peers, society)	2.2 Feedback on behavior 3.1 Social support [unspecified] 3.2 Social support (practical) 10.4 Social reward 11.2 Reduce negative emotions

##### Environmental Context and Resources

Easy access to highly palatable or “trigger” foods, coupled with the absence of a meal structure, heightened vulnerability to binge eating. Major life transitions and high-pressure home environments, especially those emphasizing achievement or appearance, were seen as amplifying these vulnerabilities. Participants noted that family members often underestimated how such environmental cues could contribute to symptom escalation: “If certain foods are in the house, it’s like I can’t stop myself- they just feel too tempting” (FG B, Participant 2).

Family dynamics and expectations played a critical role in shaping disordered patterns. For some, distancing from high-pressure environments provided temporary relief and space for recovery. Although many participants valued the structure and accountability that family meals offered, these situations were also commonly experienced as pressurizing. Expectation around portion size, pace of eating, or food type frequently triggered conflict and, in some cases, intensified ED behaviors.

##### Social Influences

Participants emphasized the powerful role of social influences in shaping their eating behaviors, spanning both protective and detrimental effects. Supportive relationships with peers, family, and professionals who understood their experiences were viewed as critical in reducing isolation and fostering accountability, illustrating how social understanding could reinforce recovery motivation:

Having someone who understood what I was going through made a huge difference. It gave me the strength to keep going.

FG B, Participant 1

Online communities generated mixed experiences. While some participants valued the sense of belonging they provided, others described difficulties in maintaining safe boundaries and moderating harmful content: “If they share it [a negative eating episode] in a group chat, it may be a trigger for others getting worse, not better” (FG B, Participant 2).

Beyond interpersonal influences, exposure to idealized or contradictory nutrition content on social media was described as particularly harmful, shaping unrealistic self-comparisons and reinforcing internalized dietary norms. One participant rued: “Looking at dieting stuff and following people who I admired when I should not have admired them” (FG F, Participant 2).

#### Reflective and Automatic Motivation

##### Overview

Key reflective and automatic motivation factors influencing disordered eating behavior are summarized as themes, subthemes, and mapped behavior change techniques across COM-B and TDF domains (Table 3).

**Table 3:** Reflective and automatic motivation factors influencing disordered eating behavior: capability, opportunity, motivation—behavior (COM-B) and theoretical domains framework (TDF) analysis.

COM-B domain	TDF domain	Subthemes	Behavior change technique
Reflective motivation	Beliefs about capabilities	Low confidence that can break the cycle of eating disorder (ED) Lack of self-belief, not good enough Reflecting on successes, self-belief	1.2 Problem-solving 13.4 Valued self-identity 15.1 Verbal persuasion about capability 15.4 Focus on past success
Reflective motivation	Beliefs about consequences	Beliefs about the challenges posed by giving up	5.6 Information about social and emotional consequences 11.2 Reduce negative emotions
Reflective motivation	Goals	Action planning Goals or milestones (within app)	1.1. Goal setting (behavior) 1.3 Goal setting (outcome) 1.4 Action Planning 5.3 Information about social and environmental consequences 9.3 Comparative imagining of future outcomes
Reflective motivation	Optimism	Hearing recovery stories Positive role models	6.2 Social comparison
Reflective motivation	Social or professional role and identify	Identity Self-esteem	13.1 Identity associated with changed behavior 13.4 Valued self-identity
Automatic motivation	Emotions	Anxiety or fear Overwhelming feelings Guilt, shame, and anger Low self-esteem Feeling isolated	11.2 Reduce negative emotions
Automatic motivation	Reinforcement	Habits or breaking habits ED behaviors reinforce themselves (a “vicious” cycle) Positive reinforcement	8.1 Prompts or cues 8.4 Habit reversal 10.4 Social reward 10.7 Self-incentive 10.8 Incentive (outcome) 10.9 Self-reward

##### Beliefs About Capabilities

Participants described a pervasive sense of helplessness and self-doubt that undermined confidence in their ability to recover. Past unsuccessful attempts reinforced beliefs that change was unattainable, often leading to withdrawal from help-seeking: “I tried all sorts of different things, and I just thought, you know, this is just gonna be my life forever” (FG A, Participant 3).

For some, recognizing the limits of self-management represented a turning point. Acknowledging that recovery required professional or external support transformed feelings of defeat into a new form of motivation: “I tried to get over it myself, but I think I got to a point where I was like, you know, I can’t do this by myself” (FG C, Participant 3).

Building self-efficacy and challenging entrenched beliefs of inadequacy were described as essential to sustaining recovery. Structured therapeutic activities that encouraged reflection on sources of self-worth beyond appearance supported this process: “(if) you had to split up a pie chart into where you got your self-esteem from ... all these things would still be here, even if you gained weight ... you’d still be clever” (FG B, Participant 1).

##### Beliefs About Consequences

Participants demonstrated clear awareness of the emotional and physical harms linked to their ED behaviors, but often failed to translate this to behavioral change, reflecting an ongoing tension between awareness and action. Fear of the challenges associated with recovery, particularly the perceived loss of a familiar coping mechanism, was frequently described as a key barrier to change: “I know bingeing makes me feel terrible afterward, but the idea of stopping feels impossible sometimes” (FG E, Participant 1).

##### Goals

Setting short-term, achievable goals, such as planning meals or managing specific triggers, helped participants reintroduce structure and experience a sense of progress in their recovery. These goals were most effective when integrated into daily routines and aligned with personal values, as this reinforced agency, motivation, and ownership of their recovery process: “It was about setting goals that fit within my life ... it made me feel fulfilled and motivated to pursue more” (FG C, Participant 2).

##### Social and Professional Role and Identity

Participants described profound challenges in reconciling their sense of self, including their personal and professional identities, with their disordered eating behaviors. This identity conflict often created internal tension that undermined recovery efforts: “It was hard to distinguish me and the eating disorder in a way” (FG E, Participant 1).

The internalization of ED thoughts was frequently described as an “ED voice,” illustrating the extent to which the disorder became entangled with their identity. Learning to externalize this “voice,” and recognize it as separate from the self, was viewed as a critical step in reclaiming agency and distinguishing authentic identity from disordered cognition:

recognizing that this is like an actual disorder that’s trying to make me do these things like this isn’t me. I’m not the ED.

FG F, Participant 2

Social and professional roles also influenced help-seeking. Expectations of competence and self-sufficiency intensified the stigma and reluctance to seek support:

Admitting I had an eating disorder felt like a personal failure. It was hard to align my professional identity with seeking help.

FG A, Participant 3

##### Optimism

Optimism was a key driver, with participants expressing how positive role models and hearing recovery stories, particularly from individuals they could personally relate to, strengthened belief in the possibility of change and reinforced hope for recovery: “If you can show me that this has worked for people like me, I’ll believe it’s possible” (FG F, Participant 2).

##### Emotions

Emotional regulation was significant with stress, anxiety, and boredom commonly described as triggers for both binge eating and restrictive behaviors. These functioned as antecedents to maladaptive coping strategies, offering momentary distraction or relief, but ultimately perpetuating a cycle of distress fuelled by guilt and shame:

When I feel stressed, I eat to distract myself. It’s like a temporary fix, but it always makes me feel worse afterward.

FG E, Participant 1

Over time, these self-regulatory behaviors became deeply ingrained, making them a more automatic response to emotional discomfort and a barrier to developing healthier coping strategies. One participant reflected on emotional resilience: “Until you learn to ride the wave of your emotions, it’s really hard to get further because you’re still retreating to that coping mechanism” (FG B, Participant 1).

Awareness of the harms associated with disordered eating was common, but insufficient to motivate change. Usually, it was fear, associated with a pivotal moment such as a health scare, which motivated individuals to seek help: “I was so scared by the symptoms that I was having that I felt I had to do something about it” (FG A, Participant 1).

##### Reinforcing Behavior

Disordered eating patterns were often described as habitual responses to distress, reinforced by their short-term soothing effects. Participants emphasized the importance of shifting these ingrained behaviors by introducing more constructive and compassionate coping strategies. Reframing from punishment to positive reinforcement was seen as key to breaking the cycle and enabling more sustainable change: “When I started focusing on rewarding myself for healthy habits, instead of punishing myself for slip-ups, it really helped” (FG B, Participant 3).

Encouragement, both internal and external, played a vital role in reinforcing healthier behaviors. Conversely, the absence of positive feedback could undermine progress and deepen feelings of failure: “You feel rubbish and that just perpetuates the disorder” (FG A, Participant 1).

#### Demographic Differences

##### Male Perspectives

Although many experiences overlapped across both sexes, male participants identified distinct influences on their EDs, shaped by societal expectations, stigma, and masculine identity norms that often inhibited help-seeking: “Masculinity plays such a big role, it’s hard to even admit you’re struggling with something like this” (FG D, Participant 1).

Cultural ideals of the “ideal male body” were described as emphasizing leanness and muscularity rather than thinness, leading to restrictive eating and compulsive exercise. This pursuit of muscularity was reinforced by comparison with others in fitness environments and online spaces, heightening dissatisfaction and reinforcing unhelpful behaviors:

When you’re constantly seeing guys at the gym who are shredded, it makes you feel like you’re not good enough. You start to think you have to eat and train in a certain way to get there.

FG D, Participant 3

These pressures led to rigid control over diet and exercise, including avoiding foods perceived as “unhealthy” and obsessively monitoring calories. Limited understanding from family members often left male population bottling up struggles, increasing shame and isolation. A related challenge was the lack of male representation in ED discourse, which left participants feeling invisible and reinforced the perception that EDs were a “female issue.” This invisibility was compounded by experiences of seeking support, which were often delayed or met with misunderstanding:

You feel like eating disorders are seen as a “female issue.” When men reach out, it’s often too late, or they’re misunderstood.

FG D, Participant 4

Despite these barriers, male participants identified adaptive coping strategies, including grounding techniques, engaging in hobbies, and goal setting. Distraction and hobbies were helpful: “I focus on grounding myself, thinking about the bigger picture, and using distractions like video games or my favorite music” (FG D, Participant 1).

The value of peer connection, particularly with other male participants, was emphasized as a powerful way to normalize experiences and promote recovery-oriented conversations: “knowing there are other guys out there going through the same thing ... it makes you feel like you’re not alone in this” (FG D, Participant 4).

##### Ethnic Minority Perspectives

Ethnicity emerged as a significant factor influencing ED experiences across both male and female participants, although the impact of cultural norms varied by sex. Participants from non-Western cultural backgrounds described strict societal standards regarding body weight and appearance. These pressures were often more pronounced for female participants, with body size and weight being closely tied to social value:

I wear a medium size in the UK, but in China, I wear XXL. It makes girls feel like they’re fat even when they’re not.

FG E, Participant 2

Big girls are seen as less likely to get married.

FG E, Participant 2

Cultural practices, such as food-oriented community events (eg, Chinese New Year), were described as intensifying disordered eating behaviors:

During holidays, it’s all about food, and they keep asking me to eat, eat, eat. It’s very hard for someone trying to manage their eating.

FG B, Participant 2

Limited family understanding of EDs was reported across participants. However, female participants more frequently highlighted the direct impact of family comments on self-esteem and body image, whereas male participants emphasized the challenge of navigating EDs within culturally normative and family context. Cultural stigma posed barriers for both male and female participants but manifested differently. Female participants described cultural attitudes that trivialized EDs as vanity, while male participants encountered stigma framing these conditions as inherently feminine or a sign of weakness, impeding recognition and help-seeking: “Mental health issues are often ignored in my culture, and eating disorders for men aren’t even seen as a real thing” (FG D, Participant 5).

Participants noted tension between Western beauty ideals and their cultural norms, with exposure to unattainable media-driven body standards contributing to heightened body dissatisfaction: “The media promotes certain body types that are unattainable for most of us, especially people from different ethnic backgrounds” (FG D, Participant 3).

### Objective 2: Influences on App Engagement

#### Overview

The analysis of app engagement against the COM-B and TDF revealed that all COM-B components, except physical capability, played a significant role in influencing app engagement. These components aligned with 12 of 14 (86%) TDF domains, highlighting key mechanisms of change and implications for app design.

#### Psychological Capability

##### Overview

Key psychological capability factors influencing app engagement were identified, with associated themes, subthemes, and behavior change techniques across COM-B and TDF domains (Table 4).

**Table 4:** Psychological capability factors influencing app engagement behavior: capability, opportunity, motivation—behavior (COM-B) and theoretical domains framework (TDF) analysis.

COM-B domain	TDF domain	Subthemes	Behavior change technique
Psychological capability	Knowledge	Knowing the benefits or how will it help me?	4.1 Instructions on how to perform a behavior
Psychological capability	Behavior regulation	Reminders to use specific features in an app Set and review goals related to app use	1.5 Review behavior goals 7.1 Prompts or cues
Psychological capability	Memory, attention, and decision-making processes	Reduce cognitive overload	11.3 Conserving mental resources

##### Knowledge

Participants displayed variable understanding of how apps functioned and how they could support recovery. Those earlier in their recovery journey generally had lower familiarity of ED apps.

Some participants identified the potential for an app to learn about their condition and enhance self-awareness. Psychoeducational content relating to EDs, coping strategies, and recovery principles was consistently regarded as essential to support engagement and inform decision-making around recovery:

When I first started, I didn’t even know what eating disorders were. If an app had explained what I was experiencing and why, it might have helped.

FG F, Participant 1

Participants expressed that apps should clearly communicate their purpose, instructions, and benefits. Many expressed confusion about how existing apps should be used and questioned how certain features, such as food and thought logging, would aid their recovery: “The main reasons that I don’t like using it is because ... it didn’t really give me a very clear idea about why I am doing this” (FG B, Participant 2).

##### Behavior Regulation

Participants sought consistent engagement through behavioral prompts and tools that promote habit formation. Reminders were frequently cited as helpful:

I need reminders to log things. Otherwise, I just forget or don’t prioritize it.

FG F, Participant 1

However, overreliance on self-monitoring features, such as logging every action or thought, could make apps feel burdensome rather than supportive. Logging calories, food intake, and exercise could be unhelpful or harmful in the context of EDs: “it makes me obsessed with the calorie counting” (FG E, Participant 2).

Any self-monitoring had to avoid numbers to prevent obsessive behaviors: “Not something to measure, something to kind of beat yourself up about” (FG C, Participant 3).

##### Memory, Attention, and Decision Processes

Many participants described cognitive barriers to engaging with ED apps, noting that complex interfaces, hard-to-use “unclear” interfaces, and confusing navigation imposed demands on them beyond simply knowledge requirements. Many reported downloading apps but failing to open them or use them consistently, suggesting that early impressions strongly influenced sustained engagement:

Some apps have so many options and buttons that I don’t even know where to start. If it’s not obvious, I won’t use it.

FG A, Participant 1

Simplicity and intuitive design were highly valued, with participants avoiding ED apps with too “much stuff” on them and preferring apps that were more straightforward and easily navigable. Overly cluttered apps were abandoned, while streamlined interfaces enhanced usability and ongoing engagement: “Laying things out, so things are easier to find ... you don’t have to go searching for things and forget where it was the next time you go back to the app and CBT” (FG C, Participant 2).

Several participants suggested that delivering interactive content such as “videos, activities, and tutorials” could further support attention, comprehension, and engagement, while providing an improved user experience (UX).

#### Environmental and Social Opportunity

##### Overview

Results identified key environmental and social opportunity factors influencing app engagement, captured as themes and subthemes across COM-B and TDF domains with corresponding behavior change techniques (Table 5).

**Table 5:** Environmental and social factors influencing app engagement: capability, opportunity, motivation—behavior (COM-B) and theoretical domains framework (TDF) analysis.

COM-B domain	TDF domain	Subthemes	Behavior change technique
Environmental opportunity	Environment, context, resources	Overwhelming app “environment” Remove barriers to accessibility Avoid advertisements or pop-ups	11.3 Conserving mental resources 12.1 Restructuring the physical environment 12.4 Distraction
Social opportunity	Social influences	Befriending or peer support within the app Community or opportunity to share experiences Role models (within app) Involve professionals	3.1 Social support (unspecified) 6.1 Demonstration of the behavior 9.1 Credible source 10.4 Social reward

##### Environmental Context and Resources

Participants highlighted a lack of accessible support across the recovery journey and viewed the potential for an app to provide continuous, reliable guidance as highly valuable. Ease of access was central to engagement; participants preferred tools that integrated smoothly into their daily environments (eg, calendars) and could be opened quickly without friction: “I use my phone all the time, so if the app is easy to open and doesn’t require a lot of setup, I’d use it more” (FG E, Participant 1).

Apps were also seen as a way to bridge gaps in formal care, particularly for those who were excluded from services due to diagnostic thresholds or limited National Health Service availability. Early access through an app was considered a crucial resource for preventing symptom escalation and entrenchment of behaviors:

I wasn’t able to get help through the NHS because my BMI wasn’t low enough. If an app could offer support earlier, it would make a big difference.

FG F, Participant 2

##### Social Influences

Peer support was a consistent facilitator of app engagement. Many participants suggested that apps providing opportunities for social interaction, such as moderated support groups, could offer a sense of community and shared understanding. Connecting with others who had similar lived experiences offered companionship that professional support alone did not always provide, reducing isolation and reinforcing motivation:

When I was recovering, I didn’t feel like I had anyone to talk to who understood. An app that provided that connection would have helped so much.

FG B, Participant 1

Participants also emphasized the motivational role of seeing others’ recovery journeys. Exposure to peers’ experiences and strategies was perceived as inspiring, and it helped users to feel less isolated and able to envision positive change:

Just kind of their stories and what had helped them would help me in seeing how someone could go from this to being this person and being happy with themselves.

FG F, Participant 1

However, while social interaction was valued, moderation was viewed as essential to maintain safety and constructive engagement. Unmoderated spaces were viewed as potentially triggering or unhelpful, highlighting the need for structured guidance within peer features:

Moderation made a huge difference. In a structured group, you’re less likely to worry about things spiralling out of control.

FG B, Participant 1

#### Reflective and Automatic Motivation

##### Overview

Results identified key reflective and automatic motivational factors influencing app engagement, captured as themes and subthemes across COM-B and TDF domains with corresponding behavior change techniques (Table 6).

**Table 6:** Reflective and automatic motivation factors influencing app engagement: capability, opportunity, motivation—behavior (COM-B) and theoretical domains framework (TDF) analysis.

COM-B domain	TDF domain	Subthemes	Behavior change technique
Reflective motivation	Beliefs about capabilities	Ability to use app effectively	15.1 Verbal persuasion about capability 8.2 Focus on past success
Reflective motivation	Beliefs about consequences	App will not help Negative consequences of engagement (eg, obsessive behaviors) Frame the app as nonjudgment and supportive resource Normalize setbacks, missed logins, incomplete activities	5.2 Information about health consequences 5.3 Information about social and environmental consequences 11.2 Reduce negative emotions 11.4 Paradoxical instructions
Reflective motivation	Intentions	Support self-monitoring of behaviors	4.1 Instruction on how to perform behavior 1.1 Goal setting (behavior) 10.8 Incentive (outcome)
Reflective motivation	Optimism	Potential for positive outcomes	5.1 Information about health consequences 5.3 Information about social and environmental consequences
Automatic motivation	Emotions	Avoid negative emotional triggers Enable positive emotional responses via the app	10.4 Social reward 11.2 Reduce negative emotions
Automatic motivation	Reinforcing behavior	Positive reinforcement Minimize risk of perfectionism or fear of failure	8.1 Prompts or cues 10.3 Nonspecific reward 10.4 Social reward 10.6 Nonspecific incentive 10.9 Self-reward 10.7 Self-incentive 10.8 Incentive (outcome)

##### Reflective Motivation

###### Beliefs About Capabilities

Participants’ sense of confidence in their ability to engage with an ED app was a major determinant of whether they persisted with it. Rather than reflecting simple usability issues, uncertainty often stemmed from a deeper belief that they may not be able to use an app “properly” or benefit from it without structured support. Several participants described feeling uncertain when features lacked clear guidance or progress was difficult to track, with some questioning their own competence and disengaged as a result. Explicit guidance and opportunities for early success were described as crucial for building a sense of capability and reducing anxiety:

If the app had step-by-step instructions or guided prompts, I’d have felt more confident using it. Instead, I felt lost.

FG E, Participant 1

###### Beliefs About Consequences

Beliefs about the potential consequences of using apps significantly shaped participants’ willingness to engage. Concerns centered on data privacy and confidentiality, with fears about misuse or excessive collection of personal information creating hesitation and distrust. For some, these concerns raised questions about whether any potential benefits outweighed the risks, with “intrusive” setup questions as a barrier to use:

I’m worried about how my data will be used. Will it be sold or shared without me knowing?

FG F, Participant 2

Some expressed doubts about professional endorsement and evidence-based content, which further shaped their willingness to engage. Participants questioned whether app guidance was reliable, highlighting the importance of transparent evidence or clinical involvement:

I just don’t know if these apps are really supported by professionals. It makes you wonder if the advice is even valid.

FG C, Participant 2

Previous negative experiences with digital tools also contributed to disengagement. For some, unmet expectations led them to abandon app use or stop searching for suitable interventions:

What’s the point of using an app if it won’t really help? I don’t want to waste time on something that doesn’t make a difference.

FG D, Participant 3

###### Optimism

Although some users expressed initial skepticism about existing apps, there was cautious optimism that thoughtfully designed apps could provide meaningful support. Avoiding common pitfalls, such as calorie tracking, and emphasizing positive habits were seen as essential to success. One user suggested that providing evidence of positive outcomes from previous users’ experiences could enhance confidence in the app’s effectiveness and motivate continued use:

If you are able to work on using this app till day 30, you will be able to see 50% of people will be able to do “whatever like” positive outcomes. Then this makes me feel more hopeful.

FG B, Participant 1

###### Intentions

Participants’ intentions to engage with apps were shaped by the extent to which its features aligned with their recovery goals, personal values, and emotional needs. Certain features, particularly food tracking, were frequently described as intrusive or triggering due to associations with past disordered behaviors and often discouraged ongoing use:

When I’ve used apps that have any relation to having to log food, it’s really, really a no-no for me. I really don’t like it.

FG A, Participant 2

In contrast, some participants expressed a preference for alternative journaling options that allowed them to track thoughts or emotions. These approaches felt safer, more meaningful, and supportive of self-reflection, enabling users to identify patterns in their experiences without exacerbating disordered behaviors: “I’d rather track my thoughts or feelings than food—it feels less triggering but still helpful to see patterns” (FG A, Participant 1).

##### Automatic Motivation

###### Emotions

Emotions were pivotal in shaping participants’ engagement with apps, influencing both initial uptake and sustained use. Positive emotions, such as feelings of accomplishment, motivation, and connection, acted as key drivers of app engagement and could be achieved through supportive features within the app, helping participants feel recognized and reinforced within the app:

The milestones and rewards made me feel like I was actually making progress. Even small wins felt good and gave me a reason to come back to the app.

FG D, Participant 1

Negative emotions could act as barriers. Participants reported that features emphasizing rigid expectations or highlighting perceived failures often triggered anxiety, guilt, or frustration, reducing willingness to continue: “Missing a day felt like I’d failed, and it was really hard to come back to the app after that” (FG B, Participant 2).

Similarly, overly frequent or intrusive notifications and advertisements were described as frustrating and disengaging. Social features were perceived as emotionally supportive, helping reduced feelings of isolation and sustain motivation. Connecting with peers and observing others’ recovery journeys provided reassurance and hope:

The community aspect made me feel like I wasn’t alone. Reading about others’ experiences gave me hope and encouraged me to keep going.

FG B, Participant 1

###### Reinforcement

Participants identified the potential for apps to support the replacement of harmful ED habits with healthier, substituted behaviors, such as establishing regular eating patterns or engaging in alternative activities. Opportunities to engage in varied activities that allowed users to explore different aspects of themselves were seen as particularly valuable. Providing positive reinforcement, such as reminders of achievements, motivational quotes, or visual cues of progress, was highlighted as a key mechanism to sustain engagement especially during challenging times:

Whether that’s in forms of quotes, pictures of yourself that you have uploaded ... like almost being overwhelmed with the reminders of the good that is in your life in those moments of bad.

FG C, Participant 3

Mixed responses to generic motivational elements like milestones, badges, or standard quotes suggested that tailoring feedback to individual preferences and experiences would enhance both acceptability and effectiveness.

##### Demographic Differences

###### Male Perspectives

Male participants raised concerns about gendered design and content as a barrier to engagement. Much of the existing material within apps appeared exclusively targeted primarily at the female population:

A lot of the content seems aimed at women, like talking about menstruation or things that don’t apply to me. It makes you feel like it’s not really for you, even though you’re going through the same thing.

FG D, Participant 3

The language, visuals, and overall tone of apps were perceived as stereotypically female-centric approaches, limiting their appeal for male users. Incorporating gender-neutral design elements was suggested as a way to increase inclusivity and engagement. Their feature preferences differed from female users, with some male users highlighting the appeal of gamification and progress tracking to sustain motivation:

I loved apps that gamified progress—it kept me coming back. Milestones and achievements make a big difference.

FG D, Participant 1

Given the specific stigma surrounding EDs in the male population, participants emphasized the importance of dedicated spaces for male users. Peer support in a male-specific context was seen as reducing isolation and normalizing help-seeking:

As a guy, it’s not something you really talk about. Having a space where other men are going through the same thing makes it feel less isolating, like you’re not the only one dealing with this.

FG D, Participant 3

###### Ethnic Perspectives

Both male and female participants described available apps as often culturally irrelevant or overly Westernized, which reduced their perceived accessibility and effectiveness. The lack of diverse representation in imagery, language, and case scenarios diminished an app’s relatability, reducing the likelihood of sustained engagement:

A lot of apps feel very Western in their focus. They don’t reflect the food I eat or the challenges I face culturally.

FG E, Participant 2

Apps were also seen as insufficiently responsive to the ways cultural norms shape eating behaviors and recovery experiences. They stressed the need for diversity not only in imagery but in the perspectives and assumptions embedded in the app’s guidance:

It would help if the app showed more diversity, not just in visuals, but in the way it talks about food and eating habits. It should feel like it’s for everyone, not just one group.

FG D, Participant 5

Cultural stigma surrounding EDs was a significant barrier to help-seeking and engagement. Participants described family or community taboos around discussing EDs, emphasizing the need for apps to offer culturally sensitive approaches that normalize help-seeking: “An app that normalizes eating disorders for men [in my culture] would make it easier to reach out for help” (FG D, Participant 5).

Cultural influences shaped coping strategies and recovery needs in nuanced ways. Female participants put a greater emphasis on needing family education and support, as family expectations often directly influenced their disordered eating behaviors. Male participants highlighted the need for culturally sensitive tools that validated their experiences and helped normalize EDs for the male population, making it easier for them to open up.

## Discussion

### Key Findings

This study advanced the design of digital interventions for EDs by providing the first systematic application of the COM-B model and TDF to both the mechanisms driving maladaptive ED behaviors and app engagement. The combined analysis mapped identified behavioral determinants to 32 BCTs across 13 of the 14 TDF domains. Many of these BCTs, such as self-monitoring, feedback on behavior, and problem-solving, aligned were consistent with prior digital ED interventions [27]; however, gaps and recommendations for future development were identified (Table 7). This study extended prior work by revealing how BCT implementation was experienced by people with lived experience in digital contexts, providing insight into why theoretically sound techniques sometimes fail [39] or even have adverse effects if implemented without sensitivity to user needs. It elucidated how interactive tools, such as food logging, reminders, and progress tracking, frequently trigger shame, perfectionism, obsessive tendencies, or cognitive overload eroding reflective and automatic motivation, leading to reduced engagement and poorer treatment outcomes [39].

**Table 7:** Summary of behavior change technique coverage across capability, opportunity, motivation—behavior (COM-B) and theoretical domains framework in research-developed eating disorder (ED) apps.

COM-B domain	Coverage in commercial ED apps [25,26,54]	Coverage in evidence-based research apps [26,27,55,56]	Positive recommendations for future ED apps
Psychological capability	Very limited, mostly basic mood logging or generic tips. Little structured psychoeducation or cognitive work.	Strong. Psychoeducation, self-monitoring, reframing, goal-setting, and action-planning are well represented.	Include psychoeducation on less common or atypical EDs (eg, ARFID^a^). Avoid stereotyped or diagnosis-specific content that may not reflect individual experiences. Stage-of-change and early recovery–matched psychoeducation (especially “what is an ED?” and normalizing relapse cycles). Explicit teaching that short-term fear-food exposure does not cause large or permanent weight gain. Cognitive skills delivered via simple, nonoverwhelming formats. Qualitative or pattern-based (nonnumeric) self-monitoring only. Structured interpersonal or assertiveness scripts for disclosure and boundary-setting.
Environmental Opportunity	Almost absent, dominated by calorie or weight tracking. No environmental restructuring.	Very limited. Restricted to basic reminders or prompts.	Simple meal-planning and grocery tools. Advance planning for high-risk meals or social eating events. Stimulus-control prompts (eg, removing binge triggers). Culturally tailored food libraries. Adding in-app tools or resources (meal planners, coping cards).
Social opportunity	Rare. Occasional unmoderated forums, predominantly solitary use.	Moderate. Peer affirmations and communities exist, but practical support is minimal, and moderation could be improved.	Heavily moderated, expert-facilitated peer support, and recovery role-model content. Structured peer-mentoring or buddy systems. Guided disclosure or boundary-setting exercises. Zero tolerance for body or weight or food talk in community spaces.
Reflective motivation	Very limited. Occasional motivational quotes, no systematic identity or values work.	Strong. Goal-setting, progress review, and cognitive techniques are common.	Explicit externalization of the “ED voice” exercises. Value clarification and nonappearance-based self-esteem modules (eg, pie-chart exercises). Exposure to longer-term recovery narratives. Removal of streak counters and any visible “failure” metrics. Normalization of incomplete progress and missed days.
Automatic motivation	Limited. Mostly streak badges and generic encouragement.	Moderate. Emotion regulation, distraction, habit reversal, and social reward are frequently used.	Timer-based urge-surfing tools with progressive delay (“ride the wave” framing). Rapid-relief grounding or breathing exercises triggered by mood check-ins. Noncomparative, nonperformance-based rewards only. Graded fear-food exposure hierarchies. Shame-reducing messages after lapses or low activity.

^a^ARFID: avoidant or restrictive food intake disorder.

Psychological, social, and motivational influences on ED behaviors, such as self-esteem, identity struggles, and social isolation, emerged as central behavioral determinants, although they are often underrepresented in existing digital interventions [27]. Participants expressed a strong desire for moderated peer connection and exposure to recovery role models to reduce the intense feelings of isolation common in EDs, but only under strict moderation. This was a heightened concern in this population, reflecting the elevated risk of behavioral contagion and competitive symptom reinforcement in EDs [57]. Moderated peer support has supported engagement in digital interventions for other severe mental illnesses [58], suggesting that carefully implemented social support features could similarly enhance adherence and therapeutic benefit in ED apps. Grounding these factors within COM-B and TDF provided a structured, theory-driven framework to translate these lived experience determinants of behavior into actionable design guidance. This approach was built on previous work [38,59,60] with an ED-specific, experience-informed perspective, which was crucial, given the complex needs and vulnerability of this population.

Among strategies participants identified as interrupting disordered eating, 2 patient-generated approaches stood out: behavioral experiments in which eating previously feared foods led to the realization that weight remained stable and self-taught urge delay tactics consistently framed as “ride the wave” metaphor. Despite their therapeutic potential, these techniques remain largely absent from existing ED apps. The “ride the wave” approach has been implemented in an evidence-based app for self-harm [61], with preliminary efficacy in reducing impulsive harmful behaviors, supporting the feasibility and likely value of translating these features into ED apps.

The established importance of interface simplicity, usability, and intuitive navigation for early engagement and cognitive-load reduction was reinforced in this study, converging with existing qualitative syntheses of user preferences [16,40] and broader digital mental health engagement research [38,59,60]. However, interface simplicity was seen as a crucial requirement for this population, beyond the level typically emphasized in other mental health conditions [38,59]. This likely reflects ED-specific neuropsychological and personality traits including cognitive rigidity, impaired set-shifting [62], detail-focused processing biases [63], and clinical perfectionism [64]. A complex or unclear interface was also experienced by some as a direct threat to their self-esteem, undermining users’ confidence in their ability to use the app effectively. Perfectionism-driven shame around perceived failure within the app (eg, missing food logs or breaking streaks) further intensified disengagement.

Feeling personally understood and represented emerged as a prerequisite for engagement, particularly for male and minority ethnic participants, who often found existing apps exclusionary or irrelevant. Whereas “perceived fit” has been described as a moderator of engagement in broader digital mental health research [59], the present findings elevate it to a primary determinant of disengagement in EDs. Given that male participants comprise up to 25% of ED cases [65] and minority ethnic groups show comparable prevalence [9,66], future apps should integrate culturally specific content, reduce stigma-related barriers, and provide support spaces that are accessible and welcoming to underrepresented groups.

By delineating these ED-specific mechanisms within an established behavioral framework, this study provided actionable, ED-specific guidance for designing digital tools that are not only more engaging but also safer for individuals with EDs. Perceived safety consistently outweighed perceived benefit as a determinant of uptake and sustained use, a pattern corroborated by ED therapists in our related study [67], making the identification and mitigation of harm an overriding priority for use.

### Current ED App Landscape and Future Directions

The vast majority (>95%) of commercially available ED apps contain minimal or no evidence-based BCTs and may even promote harm (eg, unguided calorie tracking) [25,26,54,55], with few research-developed ED apps available (eg, Recovery Record and Break Binge Eating) [56]. Table 7 illustrates the extent to which commercially available and evidence-based ED apps cover the BCTs and associated COM-B and TDF domains identified in this study. Coverage is strongest for psychological capability, with automatic motivation and physical opportunity being less frequently addressed, representing target areas for strengthening ED apps.

### Implementation Sensitivity of BCTs

BCTs are not inherently beneficial: their effectiveness depends on how they are implemented. Participants described how self-monitoring and reinforcement could either support or undermine recovery depending on framing and flexibility. Rigid numeric tracking and achievement-based rewards increased compulsivity, whereas reflective journaling, pattern recognition, and intrinsic encouragement promoted autonomy and self-regulation. These insights emphasized the need for app developers to prioritize how BCTs are delivered, not just which techniques are included. Current ED apps almost universally deliver these techniques in a fixed, one-size-fits-all format. This approach is inadequate and potentially harmful, given that the same BCT can be helpful for one person yet trigger shame, obsessive preoccupation, or disengagement in another, depending on individual profile, stage of illness, cultural context, and momentary state.

Transitioning from static to adaptive delivery is crucial and increasingly feasible with existing technology [68]. By securely analyzing in-app behavioral data (eg, logging patterns, mood ratings, journal content, feature use, and timing), future apps can dynamically tailor how each BCT is presented, for example, switching from quantitative to qualitative monitoring when risk of compulsivity is detected, moderating frequency of reminders and prompts, or adapting language, case examples, and community support spaces to better reflect male or minority ethnic experiences [69]. Such responsive, data-driven personalization directly addresses the safety and engagement barriers identified and offers a practical pathway to interventions that are both more effective and substantially safer across diverse users. Robust privacy protections, transparent decision rules, and rigorous ethical oversight remain essential prerequisites before any implementation of these approaches [70].

### Strengths

As one of the first studies to apply behavior change theory, including the COM-B model and the TDF to EDs, this research bridged behavior change science with the complex challenges of designing ED interventions. This approach provided a systematic and theory-driven method for identifying relevant BCTs while ensuring that the identified techniques were rooted in an understanding of the key drivers of disordered eating behaviors. This study contributed to filling an important gap in the literature, offering a more structured and theory-informed basis for intervention development.

The inclusion of a relatively diverse sample (sex, age, ethnicity, and ED condition) enhanced the ecological validity and relevance of the findings. By engaging people in recovery, it provided a unique lens on what has been effective and meaningful in their own journeys, offering practical insights to inform intervention development. The co-design process promoted the design of interventions that are likely to be acceptable and engaging to a broad range of users as well as empowering participants as “experts by experience.”

### Limitations

The sampling strategy and size, while sufficient for qualitative exploration and reaching a point of saturation, may have only partially captured the diversity of experiences within this population despite efforts to broaden reach and inclusivity. The inclusion of self-reported data from participants in recovery could introduce recall bias; however, their reflections were used alongside individuals still struggling with ED behaviors. Our form-based screening relied on participants’ self-reported diagnosis, treatment history, and ED behaviors, as a formal clinical diagnosis was not required. While this approach was consistent with our aim to center lived experience, it introduced the possibility of inaccuracies in participants’ diagnostic status.

An unexpected limitation of our recruitment was including a public study email address in social media advertisements, which likely increased bot submissions (~79% exclusion rate). We recommend that future studies avoid listing email addresses in recruitment materials, instead using secure, anonymized survey links (eg, REDCap and Qualtrics). Additionally, we suggest incorporating attention-check or instruction-following items (eg, “Begin your response with ‘selected codeword’”) within screening forms to further enhance data quality in online qualitative research.

Finally, the identification of BCTs in this study reflected a focus on subjective preferences and perceptions of effectiveness. It is important to evaluate the efficacy of the identified BCTs in changing ED-related behaviors at a later stage.

### Further Research

A critical next step involves effectively translating the shortlisted BCTs into an intervention design that resonates with users (Table 7) and is validated by therapists. Co-design with individuals with lived experience of EDs is fundamental, ensuring that the intervention is both relevant and accessible to a range of users. Future research should evaluate the effectiveness, acceptability, and usability of digital apps, which integrate these BCTs and offer a personalized UX. Exploration of how BCTs can be tailored to diverse user profiles, considering variations in symptomatology, age, sex, cultural background, as well as comorbidities, is also required. Developers should work collaboratively with diverse people with lived experience to test and refine how BCTs are implemented, such that self-monitoring tools or rewards do not inadvertently exacerbate stress, anxiety, or ED symptoms [16,31,62].

Evaluation will require the use of mixed methods studies to capture both quantitative measures of efficacy and qualitative feedback on UX. These BCTs should be evaluated in randomized controlled trials, exploring how their integration with cognitive behavioral therapy–based therapy impacts ED recovery rates and user engagement.

Finally, research should focus on exploring innovative personalization strategies, such as just-in-time adaptive interventions and artificial intelligence (AI), while ensuring ethical and user-centered design [70]. This includes the co-design and development of user-facing and backend AI tools to support personalization of the user journey. The potential of blended approaches that combine digital tools with therapist involvement could be explored to address deeper psychological and identity-related challenges.

### Conclusions

This study delivered the first COM-B or TDF-mapped blueprint of the psychological, social, environmental, and motivational drivers of maladaptive ED behaviors and sustained app engagement derived from people with lived experience. By emphasizing which BCTs to include and understanding how their implementation determines whether they support or undermine recovery, this study provided updated guidance for co-designing engaging, emotionally safe, and effective apps. Future interventions must integrate behavioral science, trauma-informed UX, ethical AI, and clinical expertise with ongoing involvement of diverse people with lived experience to create digital tools that are effective, engaging, and equitable across sex and ethnicity.

## Data Availability

The datasets generated or analyzed during this study are not publicly available to protect participant confidentiality, but are available from the corresponding author on reasonable request.

## Appendix

Multimedia Appendix 1 COREQ checklist.

Multimedia Appendix 2 Bot-detection protocol and exclusion breakdown.

Multimedia Appendix 3 Discussion guide for focus groups.

Multimedia Appendix 4 Baseline characteristics.

## Abbreviations

AI: artificial intelligence
BCT: behavior change technique
COM-B: capability, opportunity, motivation—behavior
COREQ: Consolidated Criteria for Reporting Qualitative Research
DSM-5: Diagnostic and Statistical Manual of Mental Disorders (Fifth Edition)
ED: eating disorder
FG: focus group
REDCap: Research Electronic Data Capture
TDF: theoretical domains framework
UX: user experience
