# Supplementary material for: Understanding Behavioral Influences on Eating Disorders and App Engagement to Inform Eating Disorder App Development: Qualitative Online Focus Groups With Adults With Lived Experience
Source: JMIR Form Res. 2026 Jan 28;10:e79328. doi: 10.2196/79328 (PMC12895151; doi:10.2196/79328)
Supplement: Multimedia Appendix 2 [file formative_v10i1e79328_app2.docx]

**Protocol Overview** Inauthentic submissions were identified using four sequentially applied criteria based on email metadata and content:

1. **Timestamp clustering**: ≥3 emails received within a 60-second window.
2. **Near-identical email addresses**: Variations differing by ≤2 characters (e.g., edstudy01@gmail.com, edstudy02@gmail.com).
3. **Verbatim or near-verbatim content**: Email bodies sharing ≥80% identical phrasing (manual review).
4. **Linguistic anomalies**: Markedly poor grammar, spelling, or nonsensical/repetitive text unrelated to eating disorders.

**Table S1. Exclusion breakdown.**

| **Criterion** | **n excluded** | **Example** | **% of total (75)** |
| --- | --- | --- | --- |
| Timestamp clustering | 22 | 7 emails in 52 seconds | 29% |
| Near-identical email addresses | 18 | user_01_study@... → user_05@... | 24% |
| Verbatim/near-duplication | 23 | 6 identical 4-sentence bodies | 31% |
| Linguistic anomalies | 12 | “ed yes yes yes eat eat yes” | 16% |
| **Total inauthentic** | **75** | — | **100%** |
