# Supplementary material for: Understanding Behavioral Influences on Eating Disorders and App Engagement to Inform Eating Disorder App Development: Qualitative Online Focus Groups With Adults With Lived Experience
Source: JMIR Form Res. 2026 Jan 28;10:e79328. doi: 10.2196/79328 (PMC12895151; doi:10.2196/79328)
Supplement: Multimedia Appendix 3 [file formative_v10i1e79328_app3.docx]

**INTERVIEW Guide for Online Focus Groups**

6 groups planned – 1 male only.

Duration: 1-hour

Median of 3 PWLE per group.

| **Section** | **Purpose / Questions** | **Notes** |
| --- | --- | --- |
| Welcome & Ground Rules | - Welcome and thank participants. - Confirm verbal consent. - Housekeeping: confidentiality, right to pass, safeguarding support. | Reassure participants that they can skip questions or leave at any time. |
| Warm-Up: Experiences & Digital Tools | - Can you share a little about your experience with an eating disorder (whatever you feel comfortable with)? - Have you used any websites or apps for support? - What did or didn’t work well? - Any early ideas of what you think could be useful in a support app? | Build early rapport while beginning to explore COM-B areas. |
| Influences on ED Behaviours | Psychological & Emotional Factors:   - What thoughts, feelings or beliefs influenced your ED behaviours? - What types of knowledge or skills have helped in your recovery? - What impact have they had (positive or negative)?   Social & Environmental Influences:   - How have people (friends, family, others) affected your ED behaviours? - Did particular places, routines, or events make things harder or easier?   Motivational Factors:   - What emotional factors influenced your ED behaviours? - What role did these behaviours play for you? - Were there any habits or automatic patterns in your behaviours? - What were your motivations or barriers to change? | Covers all domains of COM-B and TDF (e.g. beliefs, emotion, social influences, reinforcement, skills). |
| Engagement with Support Apps | - What features or support would you find helpful in a recovery app? - When do you think you’d actually use it? - What might make it easier or harder to engage with an app? - How could the app experience be improved for you? | Focused on reflective motivation, environmental context, and skills. |
| Final Reflections | - Is there anything else you’d like to share that could help us better understand recovery support needs or digital tools? | Gives space for anything unaddressed or important to the participant. |
