# Supplementary material for: Understanding Behavioral Influences on Eating Disorders and App Engagement to Inform Eating Disorder App Development: Qualitative Online Focus Groups With Adults With Lived Experience
Source: JMIR Form Res. 2026 Jan 28;10:e79328. doi: 10.2196/79328 (PMC12895151; doi:10.2196/79328)
Supplement: Multimedia Appendix 4 [file formative_v10i1e79328_app4.docx]

| **Description** | **Category** | **% (n/18)** |
| --- | --- | --- |
| Gender | Female | 72% (13) |
|  | Male | 28% (5) |
| Age Bracket | 18-25 | 22% (4) |
|  | 26-35 | 50% (9) |
|  | 36-45 | 22% (4) |
|  | 56-65 | 6% (1) |
| Ethnicity | White | 72% (13) |
|  | Asian or Asian British | 17% (3) |
|  | Black, Black British, Caribbean or African | 11% (2) |
| ED Symptoms | Dietary Restriction | 78% (14) |
|  | Binge | 39% (7) |
|  | Purge | 33% (6) |
|  | Overexercise | 56% (10) |
|  | Laxative Misuse | 0% |
| Time since onset of ED | <1 year | 0% |
|  | 1-2 years | 6% (1) |
|  | 3-5 years | 6% (1) |
|  | 5-10 years | 22% (4) |
|  | 10+ years | 67% (12) |
| Treatment History (not mutually exclusive options) | Received inpatient care | 33% (6) |
|  | Received Outpatient care | 67% (12) |
|  | Currently receiving treatment | 0% (0) |
|  | On waiting list for treatment | 6% (1) |
|  | None | 28% (5) |
